# Impacts of glyphosate-based herbicides on disease resistance and health of crops: a review

**DOI:** 10.1186/s12302-018-0131-7

**Published:** 2018-01-16

**Authors:** Daisy A. Martinez, Ulrich E. Loening, Margaret C. Graham

**Affiliations:** 1 Formerly School of Geosciences, University of Edinburgh, Edinburgh, Scotland UK; 2 Ormiston Hall, Formerly Centre for Human Ecology and Department of Zoology, University of Edinburgh, EH35 5NJ Edinburgh, Scotland UK; 3 School of GeoSciences, Crew Building, The King’s Buildings, University of Edinburgh, Alexander Crum Brown Road, EH9 3JF Edinburgh, Scotland UK

**Keywords:** Glyphosate-based herbicides, Rhizosphere, Plant disease, Soil microbes

## Abstract

Based on experimental data from laboratory and field, numerous authors have raised concern that exposure to glyphosate-based herbicides (GBHs) may pre-dispose crops to damage by microbial pathogens. In this review, we distinguish and evaluate two principal pathways by which GBHs may affect the susceptibility of crops to disease: pathway 1—via disruptions to rhizosphere microbial ecology, and pathway 2—via restriction of nutrients to crops. We conclude that GBHs have the potential to undermine crop health in a number of ways, including: (i) impairment of the innate physiological defences of glyphosate-sensitive (GS) cultivars by interruption of the shikimic acid pathway; (ii) impairment of physiological disease defences has also been shown to occur in some glyphosate-resistant (GR) cultivars, despite their engineered resistance to glyphosate’s primary mode of action; (iii) interference with rhizosphere microbial ecology (in particular, GBHs have the potential to enhance the population and/or virulence of some phytopathogenic microbial species in the crop rhizosphere); and finally, (iv) the as yet incompletely elucidated reduction in the uptake and utilisation of nutrient metals by crops. Future progress will best be achieved when growers, regulators and industry collaborate to develop products, practices and policies that minimise the use of herbicides as far as possible and maximise their effectiveness when used, while facilitating optimised food production and security.

## Introduction

Since its commercial introduction in 1974 as the active ingredient of the broad-spectrum herbicide ‘Roundup^®^’, glyphosate (*N*-(phosphonomethyl)glycine) has rapidly become the most extensively used herbicide in the history of agriculture [1–4]. Glyphosate-based herbicides (GBHs) work by blocking the activity of the enzyme 5-enolpyruvylshikimate-3-phosphate synthase (EPSPS) in the shikimic acid pathway used by plants for the biosynthesis of aromatic amino acids. Disruption of the pathway prevents their synthesis, causing plant death by amino acid starvation. GBHs are purported to offer several agricultural and municipal benefits, including:

(i) *Broad-spectrum*, *systemic weed control* GBHs are effective in the control of a very wide range of plant species, including annual and perennial broadleaves and grasses, aquatic vegetation and a number of invasive species common in agricultural and amenity environments [5]. Glyphosate is rapidly translocated from leaves throughout all plant tissues and experiences little or no within-plant degradation.
(ii) *Enabling conservation tillage* GBHs are used widely in ‘no-till’ or ‘zero-till’ systems for pre-sowing and post-harvest ‘burndown’ or ‘knockdown’ applications. These applications are for herbicidal removal of a cover crop or of unwanted crop remains before planting, a practice which reduces the need for tillage, thus reducing contributions to tillage-associated soil compaction, erosion and nutrient depletion, and allowing growers to sow seeds beneath a protective cover or ‘mulch’ of decaying plant material.
(iii) *Specificity* Due to the specificity of their intended mode of action (glyphosate inhibits the activity of a single enzyme which is essential in all plants and in some microorganisms, but not in animals), some researchers consider GBHs to be relatively toxicologically benign. Many studies also report short half-lives in soil (attributed to rapid microbial degradation) and limited bioavailability and/or transport in soil due to strong sorption onto soil mineral surfaces [1, 5, 6]. As a consequence of this specificity and effectiveness, GBHs are also used extensively in urban areas for weed control on roadsides and in public parks, etc.

Although there are clear potential benefits from use in certain settings, a number of potentially deleterious side effects of GBHs are emerging. The areas of concern include the possibility that GBH use may lead to an increased susceptibility of crops to damage by microbial pathogens. In this article, we review field and laboratory experimental work reported in the literature concerning the effects of GBHs on the susceptibility of crops to disease. Specifically, our aims were to: (i) draw together current knowledge about the impacts of GBHs on crop health, (ii) distinguish and evaluate the principal ways in which GBHs may affect the susceptibility of crops to disease, (iii) identify key gaps in current understanding and (iv) make recommendations for future research directions.

We have endeavoured to select pertinent experimental studies that, taken together, build a consistent account of the principal ways in which GBHs may interfere with crops’ disease-resistance. We discuss first the key properties of glyphosate and GBHs, and their fate in plants and soils. We then evaluate experimental findings concerning herbicide-induced changes to the rhizosphere microbial community, as well as discussing the potential impacts of such changes upon crop health. Following this, we review the currently limited findings concerning the impacts of GBHs on crop mineral nutrition.

## Properties of glyphosate

### Chemical structure and mode of action

**Fig. 1:**
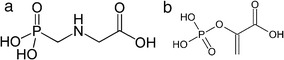
Molecular structures of **a** glyphosate and **b** phosphoenolpyruvic acid

The glyphosate molecule has three active groups: carboxylic acid (pK1 = 2.23), phosphonate (pK2 = 5.46) and amine (pK3 = 10.14) groups (Fig. 1a) and is produced, for example, by the reaction of the amino acid glycine with paraformaldehyde and dimethyl phosphonate. At pH below 2.23, glyphosate has a net zero charge, since the amino group carries one positive charge and the phosphonate group already has one negative charge; in the pH range 2.23–5.46, the carboxyl group dissociates and the molecule carries a net negative charge of one; above pH 5.46, the phosphonate group loses its second proton, the molecule then has a net negative charge of two; above pH 10.14, the amine loses its proton and the glyphosate molecule then has a net negative charge of three. The physical, chemical and toxicological properties of glyphosate are given in a technical fact sheet produced by the US National Pesticide Information Centre [7].

**Fig. 2:**
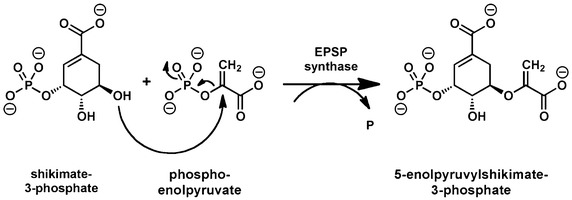
The shikimic acid pathway

These three functional groups, PO_4_^3−^, COO^−^ and R2HNH^+^, are found in many other naturally occurring molecules, but through their synthetic linkage as shown in Fig. 1a they collectively become a powerful biological agent. Glyphosate binds with and inhibits the activity of the enzyme 5-enolpyruvylshikimate-3-phosphate synthase (EPSPS) in plants’ shikimic acid pathway. In this pathway, EPSPS catalyses an unusual transfer reaction of the carboxyvinyl portion of phosphoenolpyruvate (PEP, Fig. 1b) to the 5-OH of shikimate 3-phosphate (S3P), forming EPSP and inorganic phosphate (Pi) (Fig. 2). However, the structure of glyphosate is sufficiently similar to that of PEP (Fig. 1a, b) that it can compete directly with PEP at its binding site, especially so at its binding site in the EPSPS–S3P complex [8] see also [9]. This binding reaction is not metal dependent, but the metal complexes of glyphosate (discussed below) also bind in the same way to EPSPS [10]. Among other effects, inhibition of the shikimate pathway prevents the synthesis of essential aromatic amino acids as well as a host of other aromatic plant products. Therefore, plant death can be at least partially attributed to amino acid starvation throughout all plant tissues.

### Metal-binding properties of glyphosate

In addition to its biological binding action described above, glyphosate is in an almost unique position among herbicides because of its chelating properties [11]. Indeed, it was synthesised as a chelator some 10 years before its herbicidal action was realised. Phosphonates are generally considered to be good metal chelators, but the inclusion of an amine group within the molecule usually increases the binding ability of the phosphonate. All three functional groups can co-ordinate with metal ions, particularly those of transition elements, at near neutral pH (e.g. Coutinho and Mazo [12], and an earlier study by Glass [13]. Caetano et al. [10] showed how Zn and Cu bind more extensively to glyphosate than many other metals. Indeed, copper (Cu) has been shown to form tridentate complexes with two glyphosate molecules per copper atom. More specifically, it is thought that Cu^2+^ lies at the centre of a Jahn–Teller distorted octahedron with glyphosate forming two five-member rings lying in the equatorial plane [14]. At higher pH, even tetradentate ligands can form if the phosphonate coordinates via two oxygens [11]. In contrast to most phosphonates, however, glyphosate has the unusual property of being easily precipitated out of solution by multivalent metal ions. This may reduce the acute toxic effects associated with certain metals, e.g. Ag, Cd, Cr, Cu, Ni and Pb, but some researchers have also reported increased bioaccumulation of others, e.g. Hg in aquatic organisms [15].

Given these chelating properties, it is not surprising that GBHs can exert numerous effects beyond the inhibition of target plants’ EPSPS, as will be discussed throughout this review. Potentially any metal-dependent plant metabolic processes could be affected, and thus the wider impacts could be profound.

### Distinguishing glyphosate from GBHs

Several agrochemical companies manufacture GBHs under multiple trade names. These commercial formulations contain various additives, e.g. surfactant petroleum products that facilitate their penetration of plants [16]. Thus, the impacts of GBHs on plants and other organisms may differ substantially from those of glyphosate and its salts, commonly the isopropylamine salt [17–19]. In turn, different GBH formulations have been shown to exert different effects on crops [20]. We endeavour throughout this review to cite the specific formulations used in experiments (e.g. Roundup Ultra Max^®^, Roundup Quick™). Moreover, for impacts of GBHs on crop health to be adequately risk assessed, we recommend that researchers employ commercial formulations rather than pure glyphosate in experimental protocols, since it is these that are relevant in the agricultural context.

### The fate of glyphosate in the soil

Having a systemic action, once applied glyphosate is rapidly translocated throughout plant tissues. Glyphosate residues can accumulate in newly developing plant parts, predominantly in the root and shoot meristematic tissues, but also in belowground reproductive tissues and root nodules [21]. A substantial portion of the residues that accumulate in root tissues will be released to the rhizosphere, whether due to the decay and decomposition of damaged roots or to exudation from the living roots of genetically modified glyphosate-resistant (GR) crops that have been exposed to, but not killed by GBHs [22–25]. Glyphosate applied at sub-lethal doses to non-genetically modified glyphosate-sensitive (GS) crops (where it is used to encourage uniform ripening and to ease harvesting in cereal, grain and oilseed crops) may also be destined for partial exudation from living roots [21, 24]. For example, Kremer et al. [23] reported that when GR soybean plants were treated with Roundup Ultra^®^ at a rate of 0.84 kg a.e. ha^−1^, ~ 1500 ng glyphosate had been exuded per plant after 16 days. Laitinen et al. [25] reported that 3 weeks after treatment, 8–12% of the glyphosate applied to the GS plant *Chenopodium quinoa* had been exuded from roots to the rhizosphere.

Once applied and released from roots, the binding characteristics of glyphosate and its degradation by soil microorganisms are widely reported to limit its persistence in solution and, thus, the potential to cause harm to crops [1, 6, 16]. Thus, Monsanto Company© advise growers that sowing of a new crop may commence safely as little as 4 h post-spray treatment with Roundup WeatherMAX^®^ and 24–72 h post-spray treatment with all other Roundup^®^ brand formulations [26]. Detailed evaluations indicate, however, that glyphosate residues released from plant roots cannot be assumed to be entirely or permanently immobilised, or degraded, upon contact with soil. The proportion of glyphosate residues immobilised and/or degraded has been shown to vary significantly with local soil composition and properties, as well as with climatic and meteorological conditions, and agronomic practice (reviewed in Borggaard and Gimsing [16]. Likewise, the duration of immobilisation has been shown to be variable, since already-bound glyphosate may be returned to solution (e.g. with the addition of phosphate fertilisers, which may compete with glyphosate for binding sites in soil due to chemical similarity), rendering it available once more for interactions with crops and other non-target organisms [22, 24, 27, 28] or for leaching and transfer through aquatic environments [29–33].

## The influence of GBHs on crops’ disease resistance

The following sections identify and evaluate two principal pathways by which GBHs may interfere with the disease resistance of crops.

### Pathway I: disruptions to rhizosphere microbial ecology

After release from plant roots, glyphosate residues come into contact with an immensely diverse community of microbial species dwelling in the root zone or rhizosphere. Microbial responses to glyphosate vary considerably between species. Some species possess GS forms of microbial EPSPS and suffer metabolic disruptions following exposure to GBHs [34]. Sensitive portions of the ubiquitous rhizosphere biota include some *Pseudomonas* species and some species of Mn-reducing bacteria [35–37], as well as some species of arbuscular mycorrhizal fungi (AMF) [38–40] and symbiotic N-fixing *Bradyrhizobium japonicum* bacteroides within soybean nodules [34]. The above are usually regarded as beneficial organisms. It is noteworthy that, in contrast, some potentially phytopathogenic species express relatively glyphosate-tolerant forms of the EPSPS enzyme (including some *Fusarium, Pythium* and *Rhizoctonia* spp.) and are unharmed or even stimulated in response to treatment of plants with GBHs [41, 42]. These differential impacts are of considerable concern, since they may cause alterations to microbial community dynamics that may, in turn, impact negatively upon the health and productivity of crops.

Via its contribution to biogeochemical and nutrient cycling, the soil microbial community as a whole provides a critical service in maintaining soil productivity, sustainability and resilience to perturbations [43]. The following discussion will review key experimental findings concerning herbicide-induced changes to the rhizosphere microbial community, as well as discussing the potential impacts of such changes upon crop health.

#### Stimulation of phytopathogenic microorganisms

Phytopathogenic rhizosphere-dwelling microorganisms are found to play a synergistic role in the herbicidal efficacy of GBHs, whereby intensified microbial colonisation on the roots of herbicide-treated plants has been shown to contribute to and/or hasten plant death, in conjunction with the herbicides’ intended metabolic mode of action [44–48].

Numerous studies have demonstrated that the population and/or the virulence of certain phytopathogenic species can be enhanced following treatment of plants with GBHs [23, 37, 44, 45, 47–53].

For example, in field studies conducted at multiple sites and over several years (1997–2007), Kremer and Means [37] reported that colonisation of GR soybean roots by three species of *Fusarium* (*Fusarium oxysporum* complex, *Fusarium solani* complex, and *Fusarium equiseti* complex) was significantly elevated (two- to fivefold) in plants that were treated with a GBH at recommended field rates (0.84 kg a.e. ha^−1^), compared with control plants that received no herbicide. *Fusarium* populations were also consistently elevated in soils where herbicide-treated GR crops had been grown. The authors did not specify the GBH formulation used in their tests. Similarly, when GR soybeans of two cultivars (‘BRS242’ and ‘AG3539’) were treated with a GBH (Roundup WeatherMAX^®^; Monsanto, St Louis, MO) at increasing concentrations from 800 g up to 2400 g a.e. ha^−1^, *Fusarium* colonisation of soybean roots increased significantly, in a dose-dependent fashion [36]. In a large-scale field-monitoring programme conducted across multiple cereal-cropping systems in Saskatchewan, Canada, Fernandez et al. [53] assessed the relative importance of various agronomic practices (including tillage and herbicide regimes) as factors determining inoculum levels of fungal pathogens associated with *Fusarium* head blight (FHB) (including *Fusarium avenaceum*, *Fusarium culmorum* and *Fusarium graminearum*), as well as the prevalence of FHB disease damage in wheat and barley. For both crops, the study identified glyphosate-based weed control methods as the most important management factor associated with elevated *Fusarium* spp. populations, as well as with increased disease damage in cereal crops year on year.

It has been proposed that proliferations of opportunistic microbial pathogens in the rhizospheres of GBH-treated weeds or crop residues might increase the risk of disease for new crops planted subsequently in the same soil [49–51, 54–56]. Among these, Lynch and Penn [49] found that treatment of quackgrass (*Elymus repens*) with glyphosate (isopropylamine salt) stimulated rapid colonisation of the roots of this weed by *Fusarium culmorum.* Enhancement of the rhizosphere *F. culmorum* population was associated with increased disease damage in barley seedlings planted subsequently in the same soil. An extensive field study conducted by Smiley et al. [50] showed that the inoculum potential for *Rhizoctonia solani* in cereal fields was at its peak 2–3 days after GBH treatment, before gradually declining. Yield depletion and crop damage from Rhizoctonia root rot (caused by *Rhizoctonia solani*) was most severe when the interval between herbicide treatment of unwanted vegetation and direct drilling of spring barley was shortest, e.g. 2–3 days from herbicide treatment to spring planting. Disease damage was least when intervals between weed treatment and spring planting were longest, e.g. weed treatment in the previous autumn with crops sown in spring. Recent studies have yielded similar results; disease damage to spring seedlings of onion [55] and corn [56] was minimised when the time interval between GBH application to cover crops of winter cereals and planting of the spring crop was maximised. For example, when the time interval between GBH application and onion planting increased from 3 days to 19 and 27 days, the total area of onions affected by stunting due to infection by *Rhizoctonia solani* decreased by 54–63% [55]. Taken together, the above findings are inconsistent with commercial recommendations that sowing may proceed as little as 4–72 h after herbicide treatment (see “The fate of glyphosate in the soil”).

#### Repeated exposure and shifting population dynamics

It is important to consider the longer-term implications of the findings presented in “Stimulation of phytopathogenic microorganisms” above by considering whether chronic/repeated exposure to GBHs may drive a selective shift in the rhizosphere microbial community, favouring glyphosate-tolerant microbial species over sensitive ones. This selective process could have potentially deleterious consequences for crop health where phytopathogenic species are encouraged. Although extensive research has been done on short-term microbial responses to GBHs (see “Stimulation of phytopathogenic microorganisms”), only a small number of studies have sought to observe longer-term (multi-year) effects [37, 57–59]. These studies and their findings are summarised as follows.

Collated root colonisation data from Kremer and Means [37] indicate that an annual programme of GBH treatments may have encouraged gradual expansion of the glyphosate-tolerant *Fusarium* spp. population in their experimental plots over the 10 years of their field study. Relatively low levels of soybean root colonisation were detected during years 1–4 of the study (e.g. 20–40 *Fusarium* colonies per 100 cm section of soybean root) compared with marked increases during years 5–10 of the study (100–120 *Fusarium* colonies per 100 cm section of soybean root).

Zabaloy et al. [59] compared the respiration rate (F*max*) of microorganisms present in field soils with differing management histories. A ‘pristine’ grassland soil with no previous exposure to glyphosate and two ‘no-till’ agricultural soils with 15 and 11 years of exposure to GBHs, respectively, were treated with glyphosate (Nidera, 95% technical grade) in a microcosm experiment. The soil samples had been pre-incubated for 24 h to reduce the levels of endogenous nutrient and afterwards for a further week for reasons not given. The ‘pristine’ soil exhibited a significant elevation in (F*max*) (determined by measurement of oxygen use) in response to glyphosate treatment, compared with untreated controls and with the two ‘no-till’ soils, which showed either unaffected or decreased *Fmax* following glyphosate application. The authors attributed the elevated microbial respiration rate in the ‘pristine’ soil to a stress response, inferring that a larger proportion of the soil’s microbiota may have been vulnerable to glyphosate toxicity. They suggested that increased microbial respiration may occur under glyphosate-induced stress due to the quantity of ATP diverted towards accumulation of shikimate and hydroxybenzoic acids following EPSPS disruption [59, 60]. It is questionable, however, whether such an accumulation (which is due to an inhibition and not an increased synthesis) would result in such a diversion of ATP. An alternative explanation could be that the increased respiration results from an uncoupling of oxidative phosphorylation by glyphosate.^1^ Nicolas et al. [61] provided evidence for this in fungal mitochondria. In *Aspergillus nidulans*, germination, growth and development were all inhibited by very low concentrations of Roundup, yet mitochondrial respiration was increased.

While it is not possible to determine (based on the available evidence) whether the increase in microbial respiration observed by Zabaloy et al. [59] had indeed occurred in response to glyphosate *stress*, the contrast in responses between the two samples does suggest that different management histories (i.e. chronic GBH exposure vs. no GBH exposure) had resulted in microbial communities that were either structurally or functionally different from one another, and which responded to glyphosate treatment in different ways.

Allegrini et al. [58] subsequently applied a ‘pollution-induced community tolerance’ (PICT) approach to assess whether chronic glyphosate exposure might exert selective pressure on microbial community structure and result in increased microbial GBH tolerance. The authors hypothesised that microbial tolerance to GBHs would be higher in soils with a multi-year history of GHB exposure than in soils that never encountered the herbicides. However, in contrast to the conclusions of Zabaloy et al. [59], the PICT assays found that microbial response to a GBH treatment was unrelated to a previous history of herbicide exposure (the microbial respiration rate was not significantly different between soils) and the study as a whole identified no clear evidence for localised microbial adaptation to GBHs.

Finally, a study conducted by [57] compared bacterial community composition in the rhizospheres of two crops. Corn [*Zea mays*; cultivar DKC62-54 (VT3)] and soybeans (*Glycine max*; cultivar OX 20-8 RR) were grown in a soil with no previous history of GBH exposure, within rhizoboxes. The GBH (Roundup Powermax, at recommended field rates) was applied to both crops (once prior to sowing, and twice more when plants reached V4 and V7 growth stages, respectively) during each of four 58-day cropping cycles. At the end of the fourth cropping cycle, the authors used next-generation barcoded sequencing to identify specific bacterial taxa shifts occurring in the rhizospheres of GBH-treated plants, compared with those of untreated control plants. For both corn and soybean crops, the study revealed subtle alterations to microbial community composition in response to GBH treatment. The authors observed an increase in the relative abundance of sequences associated with members of the phylum *Proteobacteria* (*p* = 0.096) in rhizospheres of both corn and soybean (e.g. from an average of 22.9 ± 1.5% in control samples to 25.9 ± 0.9% in the rhizosphere of GBH-treated corn). The increase in relative abundance of the phylum *Proteobacteria* was driven by increases in sequences from the family *Xanthomonadaceae*; the authors inferred that these may have been enriched by GBH exposure. In contrast, the relative abundance of the phylum *Acidobacteria* showed a decrease in response to GBH treatment (*p* = 0.083) in rhizospheres of both crops (e.g. from an average of 21.5 ± 1.1% in control samples to 18.7 ± 0.8% in the rhizosphere of GBH-treated corn). Since some members of the *Acidobacteria* are thought to be important contributors to biogeochemical processes within the rhizosphere, the authors suggested that a consistent decrease in their abundance over time could lead to changes in the nutrient status of the rhizosphere and could impact on the health and productivity of crops.

#### Three potential factors underlying stimulation of phytopathogens

##### Nutritional stimulation

Numerous microbial species are able to metabolise glyphosate as a direct source of nutrition [23, 64–70] Some rhizosphere-inhabiting *Fusarium* spp., for example, have been shown to metabolise glyphosate in plant root exudates as a source of phosphorus (P), carbon (C) and energy [68]. In addition, herbicide-induced changes to the composition and/or the quantity of treated plant root exudates may further enhance the nutritional richness of the rhizosphere for microorganisms poised to metabolise these products. Following treatment of two soybean cultivars (GR; ‘Pioneer 94B01’, and GS; ‘Williams 82’) with a GBH (Roundup Ultra; Monsanto, St Louis, MO) at a rate of 0.84 kg a.e. ha^−1^, Kremer et al. [23] found that glyphosate residues were exuded from roots of both cultivars in steadily increasing concentrations from 2 up to 12 days after herbicide treatment. From 12 to 16 days after treatment, glyphosate exudation continued at a near-constant rate from the living roots of the GR cultivar, but diminished in the GS ‘W82’ cultivar, apparently slowed by the death of the plants. In addition, it was observed that root exudates of treated soybeans (both GS and GR) contained high concentrations of free amino acids and soluble carbohydrates, compared with the exudates of untreated controls. These products are rich nutrient sources for bacterial and fungal phytopathogens, as well as insect pests. When cultured directly in the root exudates of treated plants, select *Fusarium* spp. strains (strains 301 and 304) developed significantly higher microbial biomass compared with controls [23]. Likewise, Liu et al. [71] found that germination and growth of *Pythium ultimum* germ tubes was significantly enhanced when isolates of the pathogen were cultured in root exudates of Roundup-treated bean seedlings, compared with those of untreated controls.

Neither of the two studies [23, 71] was able to distinguish the stimulatory effects of exuded carbohydrates and amino acids from those of exuded glyphosate. Nevertheless, it appears that the combined release of residual glyphosate, along with elevated quantities of soluble metabolic products from the roots of GBH-treated plants, may have been of direct benefit to certain opportunistic phytopathogens present in the rhizosphere. This is consistent with Chaboussou’s theory of trophobiosis [72] and references therein (see below), which states that phytopathogenic microorganisms proliferate where these less complex metabolic products (i.e. free amino acids and reducing sugars such as glucose as opposed to complex proteins and carbohydrates, respectively) are available in excess.

##### Impairment of the physiological defence mechanisms of crops

Since key components of the physiological disease resistance of crops, e.g. the production of various defensive metabolites and the protective lignification of cell walls, are dependent on the products of the shikimic acid pathway, exposure to glyphosate (even at sub-lethal doses) has the potential to impair innate disease resistance in plants [46, 71, 73].

Inhibition of EPSPS restricts the biosynthesis of key phenolic defence products such as antibiotic compounds, pathogen-induced anti-microbial phytoalexins, and cinnamic acid-derived lignin for structural cell wall enhancement at infection sites, [6, 42]. The inhibition of EPSPS by GBHs has been shown to inhibit the formation of defensive metabolites to the extent that the innate resistance of a crop to a pathogen is lost, or weakened [42, 47, 54, 74–76].

Early laboratory studies demonstrated that sub-lethal doses of glyphosate prevented accumulation of the defensive phytoalexin *glyceollin* in GS soybean [*Glycine max* (L.) Merr. ‘Harosoy 63’], resulting in increased susceptibility of the previously resistant soybean cultivar to infection by two pathogenic fungi, *Phytophthora megasperma* f. sp. glycines and *Pseudomonas syringae* pv. *glycinea* [73]. Sub-lethal doses of glyphosate applied to seedlings of two *Fusarium oxysporum*-resistant GS tomato cultivars were shown to severely undermine the innate resistance of tomato plants to the pathogen, resulting in severe *F. oxysporum* colonisation of tomato root tissues [76]. Sharon et al. [46] reported a substantial (fivefold) decrease in the concentration of *Alternaria cassia* conidia required to kill seedlings of *Senna obtusifolia* L. when a sub-lethal dose of glyphosate was applied in combination with the fungal inoculum, and Liu et al. [71] reported that when bean seedlings (*Phaseolus vulgaris* L.) were treated with Roundup^®^ 2 days before inoculation with mycelial suspensions of *Pythium ultimum*, defensive pathogen-induced lignification in plant roots was significantly suppressed compared with controls, rendering treated plants significantly more vulnerable to *Pythium ultimum* damage.

The collective results of these studies indicate clearly that exposure to GBHs, even at sub-lethal doses such as may occur by accidental drift, can result in significant inhibition of GS crops’ physiological defence mechanisms and thereby increased disease damage of crops.

Both Cerdeira and Duke [77] and Duke et al. [6] suggested that since GR crops express a glyphosate-tolerant form of EPSPS, herbicide-mediated impairment of plant defences via disruption to phenolic metabolism is unlikely to occur in Roundup Ready^®^ cultivars. This reasoning is logical if it is assumed that the GR EPSPS enzyme functions equivalently to the wild-type EPSPS enzyme in the absence of herbicide, and is equally efficacious in supporting disease-resistance mechanisms when exposed to herbicide. A few studies have indicated, however, that some GR cultivars are also vulnerable to inhibition of physiological defences following GBH application at sub-lethal doses [47, 78, 79].

Larson et al. [78] demonstrated herbicide-induced loss of resistance in the previously *Rhizoctonia* root rot-resistant GR sugar beet cultivar, ‘B4RR’. GBH-treated (Roundup WeatherMAX; Monsanto Co., St Louis, MO, at a rate of 0.84 kg a.e. ha^−1^) sugar beet suffered significantly enhanced disease damage when inoculated with an isolate of *Rhizoctonia solani* to which it would ordinarily be resistant, compared with untreated control plants. The authors interpreted the observed loss of resistance to be a ‘plant-mediated’ phenomenon, since in vitro *R. solani* growth assays showed no significant alterations in the growth rate of fungal isolates when cultured with the herbicide at equivalent concentrations, compared with controls. Larson et al. [78] took repeated measurements of shikimic acid accumulation (a reliable marker for EPSPS disruption) in GR sugar beet tissues at regular time intervals (0, 3, 7 and 14 days following glyphosate application). Seedlings showed significantly higher shikimate accumulation in all tissue types, except roots, and at all sampling time points in glyphosate-treated plants compared with controls. The authors proposed that the observed disruption of sugar beet EPSPS following glyphosate exposure (indicated by the increased levels of shikimate) might have been sufficient to disrupt physiological defence mechanisms, increasing the susceptibility of the plant to infection by the *R. solani* pathogen.

The findings of Larson et al. [78] are consistent with those of Sanogo et al. [47], who found that when two previously *Fusarium solani*-resistant GR soybean cultivars (Asgrow 3701, and Asgrow HIG3071) were inoculated with two isolates of *F. solani* (isolates ‘Monticello’, and ‘Scott’), glyphosate treatments (Roundup Ultra, at 0.84 kg a.e. ha^−1^) resulted in statistically significant increases in the severity of *sudden death syndrome* (measured as % *Fusarium* damage to leaves and roots) occurring in plants of both cultivars, as well as significantly increased isolation frequency of *F. solani* sp. *glycines* from plant roots. Conidial germination, mycelial growth and sporulation of *F. solani* isolates (‘Monticello’, and ‘Scott’) were reduced when glyphosate was added to the culture medium, compared with controls [47], indicating that the enhanced disease development observed *in planta* had occurred due to herbicidal suppression of plant immunity, rather than to direct stimulation of the pathogens.

Finally, a greenhouse study by Zobiole et al. [79] found that when GR soybean plants (*Glycine max* L. Merr. Cultivar BRS-242 GR) were treated with glyphosate (isopropylamine salt), soybean lignin production decreased markedly with increasing glyphosate application rates. The total lignin content of plants decreased from ~ 0.50 g plant-1 in untreated soybeans, to ~ 0.14 g plant-1 in soybeans treated with glyphosate at a rate of 1800 g a.e. ha^−1^. The authors also reported a significant decrease in the total amino acid content of treated soybean plants relative to controls, but we question the units presented with this data. Since lignin biosynthesis is dependent on phenylalanine, a key amino acid product of the shikimate pathway, the authors proposed that the observed declines in lignin production might have been due to herbicide-induced EPSPS disruption, even in their GR soybeans. Possibly, declines in soybean photosynthetic rate and chlorophyll content with increasing glyphosate application rates may also have contributed to the observed suppression of lignin biosynthesis. The authors do not comment on any reduction in protein synthesis [79].

##### Suppression of pathogen antagonists

A third factor may be the GBHs suppression of beneficial rhizosphere-dwelling microbial species which antagonise the pathogens. As mentioned above, certain species express glyphosate-sensitive forms of microbial EPSPS and suffer metabolic disruption when exposed to GBHs.

Several studies have reported declines in rhizosphere-inhabiting populations of fluorescent *Pseudomonas* spp. (ubiquitous soil bacteria capable of synthesising various defensive metabolites and antibiotics, and enhancing nutrient availability), following GBH applications to GR soybeans. For example, during the course of their long-term field study, Kremer and Means [37] reported significant decreases in rhizosphere-populations of fluorescent *Pseudomonas* spp. following GBH treatment of GR soybeans. *Pseudomonas* decline correlated with the observed increases in root colonisation by *Fusarium* spp., and bioassays of single cultures confirmed that ~ 85% of the identified *Pseudomonas* spp. were potentially antagonistic towards *Fusarium* spp. Correspondingly, the aforementioned study by Zobiole et al. [36] reported a decline in rhizosphere-inhabiting fluorescent pseudomonads following application of Roundup WeatherMAX to soybeans (GR1; BRS242 and GR2; AG3539).

A detailed analysis by Aristilde et al. [35] illustrated the significant variation that exists between rhizosphere-dwelling *Pseudomonas* species and even between strains, in degrees of sensitivity to glyphosate at varying concentrations. In sensitive species, glyphosate was shown to significantly inhibit the biosynthesis of the essential amino acids phenylalanine, tyrosine and tryptophan via disruption to bacterial EPSPS [35].

Herbicide-induced suppression of sensitive *Pseudomonas* spp. has the potential to impact detrimentally on the disease resistance of crops by: (i) reducing the availability of *Pseudomonas* spp.-derived secondary metabolites which are contributors towards plant disease defences and (ii) reducing the competitive antagonism provided by *Pseudomonas* spp. towards phytopathogenic species such as *Fusarium,* allowing these to proliferate. Fernandez et al. [53, 80] also detected GBH-induced alterations to competitive relationships between fungal species, since weed control with GBHs appeared to have a stimulatory effect on *Fusarium* spp. (causal agents of *Fusarium* head blight, root and crown rot, and sudden death syndrome) while suppressing *Cochliobolus sativus* (causal agent of common root rot, a common disease of wheat and barley). The authors inferred that the observed suppression of *Cochliobolus sativus* populations might have contributed to the relative proliferation of *Fusarium* spp. populations, due to a reduction in competitive antagonism.

### Pathway II: impacts on plant mineral nutrition

Balanced nutrition is essential for the regulation of plant metabolic processes supporting physiological disease resistance [42, 81], and crops are more susceptible to disease when their nutritional balance is not optimal, i.e. when nutrients are not available in their optimal ratios [72, 81]. GBHs have been shown to interfere with the uptake, in-plant translocation and utilisation of essential elements (e.g. metals) within crops, including those exposed to sub-lethal doses, and even to concentration levels associated with herbicide drift [24, 82–84]. Researchers have raised concerns that disruptions of this kind may compromise the innate disease resistance, elevating the risk of disease in cropping systems where GBHs are used for weed control [42]. The potential for such a relationship warrants closer scrutiny. Accordingly, this section outlines the limited published studies that examine the effects of GBHs on crop nutrition, first in the context of GS crops and then with regard to GR crops.

#### Nutritional disruption in GS crops

Eker et al. [83] demonstrated that root uptake of both radiolabelled manganese (^54^Mn) and iron (^59^Fe) was markedly reduced (~ 25 and ~ 75%, respectively) compared with controls, following treatment of sunflower seedlings with sub-lethal concentrations of a GBH (Roundup Ultra, Monsanto co., at 6.0% label-recommended dosage). Additionally, herbicide treatments resulted in near-complete inhibition of root–shoot translocation of ^59^Fe and ^56^Mn, at 12 and 24 h post-treatment, respectively. Both uptake and translocation of radiolabelled zinc (65Zn) also decreased, although not statistically significantly. Cakmak et al. [82] raised seedlings of GS soybean in soils amended with a standard complement of macro- and micronutrients. At the V4, V6, and early R1 growth stages, seedlings were treated with a GBH (Roundup Ultra, Monsanto Ltd, Turkey) at sub-lethal concentrations representing between 0.3 and 1.2% of the label-recommended dose. At soybean maturation, concentrations of calcium (Ca), magnesium (Mg), Mn and Fe were each reduced in the beans produced by herbicide-treated plants, in a dose-dependent manner. Reductions in nutrient concentrations were most extreme (e.g. Ca, Mg, Mn and Fe by ~ 26, ~ 13, ~ 45 and ~ 49%, respectively) when plants were exposed to the highest herbicide dosage (1.2% of the recommended field rate). Finally, the findings of Ozturk et al. [84] indicate that high sensitivity of the enzyme Fe-reductase to very low concentrations of GBH may play a role in glyphosate-mediated impairment of Fe^−^ uptake. They [84] investigated the effects of drift concentrations of a GBH (Roundup Ultra, Monsanto Ltd, Turkey, at concentrations corresponding to 1, 3 and 6% of the recommended dose for weed control) on the activity of ferric (Fe^3+^) reductase in root tissues of Fe-deficient sunflower seedlings. Glyphosate exposure inhibited root Fe-reductase activity in a dose-dependent manner. At the highest dose (1.89 mM, corresponding to 6% of recommended herbicidal dose), Fe-reductase activity was inhibited by 50% at 6 h after treatment and by almost 100% at 24 h after treatment.

The above results suggest a significant influence of GBHs on the uptake of some micronutrients, their root-to-shoot translocation and, in the case of the study by Cakmak et al. [82], on their accumulation in mature GS soybeans. Importantly, these findings demonstrate the potential for nutritional disruption of this kind to occur following exposure of GS plants to a small fraction of the recommended herbicidal dosage, such as may occur by accident due to spray drift or root contact with herbicide residues in soil. Mechanisms underlying the observed antagonism between GBHs and crop nutrition were not conclusively identified by these studies. It has been suggested, however, that the observed declines in uptake, as well as in-plant transport of micronutrients, might be related to the ability of glyphosate to form poorly soluble glyphosate–metal chelates within plant tissues and/or within the rhizosphere [82, 83]. Ozturk et al. [84] inferred that the formation of glyphosate–Fe complexes might have restricted the availability of Fe(III), crucial for the maintenance of Fe-reductase enzyme activity, in plant cells.

Since glyphosate binds with metal ions (see “Metal-binding properties of glyphosate”), the formation of glyphosate–metal chelates in spray solutions has been shown to limit penetration and translocation of glyphosate within plants [85–87]. In turn, it is possible that formation of glyphosate–metal chelates where glyphosate accumulates in root tissues [21] and/or in the rhizosphere [23] may restrict the availability of nutrient metals for in-plant transport and use [24, 82, 83]. Alternatively or in addition, it is possible that the nutritional disruption observed may have occurred due to low-level herbicide toxicity from the accumulation of glyphosate (and/or its primary breakdown product, AMPA) in crop roots, where partial impairment of GS EPSPS could have impaired the physiological processes associated with nutrient acquisition [6].

#### Glyphosate–nutrient antagonism in GR crops

There are conflicting conclusions in the literature about whether GBHs might impact adversely upon mineral nutrition in GR crops. A detailed review by Duke et al. [6] acknowledges that since glyphosate alters almost every physiological and biochemical process during the course of its herbicidal action in sensitive plants, it follows that the nutrition of GS crops is likely to be compromised following application of a GBH at recommended concentrations. In contrast, they also propose that GBHs should not be expected to disrupt nutritional physiology in resistant GR cultivars, due to the engineered resistance of these crops to EPSPS disruption. Indeed, several studies have detected no deleterious impacts of GBHs on GR soybean nutrition [88–91]. Among these, Duke et al. [91] detected minimal and inconsistent effects of a GBH (Roundup WeatherMAX, applied at 0.87 kg a.e ha^−1^) on foliar and seed content of Al, As, Ba, Cd, Co, Cr, Cs, Fe, Cu, Fe, Ga, K, Li, Mg, Mn, Ni, Pb, Rb, Se, Sr, TL, U, V and Zn, in GR soybeans (cultivar USG Allen GR). There were also no effects of GBH treatment on soybean grain yield [90].

However, a small number of studies have reported significant disruptions to GR crop nutrition, as well as declines in plant biomass production, chlorophyll content and photosynthetic rate, associated with GBH treatments at the recommended herbicidal dosage to GR crops [20, 79, 92–96]. These studies and their main findings are summarised as follows.

Serra et al. [93] found that accumulation of Fe, Zn, Mn and Cu was significantly reduced in GR soybeans (cv. P98R31 RR) at the V8-growth stage, when these were treated with glyphosate (formulation unspecified) at increasing rates (0, 648; 1296; 1944 and 2592 kg a.e. ha^−1^), compared with controls. Zobiole et al. [94] grew GR soybeans of three cultivars (BRS242 GR, BRS245 GR and BRS247 GR, representing early, medium and late maturity groups, respectively) in two soil types (a Typic Hapludox, and a Rhodic Ferralsol). At the V4 and V4 + V5 growth stages, soybeans were treated with either one (1200 g a.e. ha^−1^) or two (600 + 600 g a.e. ha^−1^) applications of glyphosate (as the isopropylamine salt), respectively. When measured at the R1 growth stage, leaf concentrations of Mn, Fe, Cu and boron (B) were each significantly reduced in herbicide-treated plants of the early and medium maturity cultivars. Leaf concentrations of Zn were also significantly reduced in herbicide-treated plants of the early maturity cultivar, as were the macronutrients phosphorus (P), potassium (K), Ca, Mg and sulphur (S). Only P and Mn concentrations were reduced significantly in the late maturity cultivar, compared with controls. The authors inferred that the later maturing cultivar might have benefitted from a longer period of recovery post-treatment, compared with the early and medium varieties that showed greater impacts. In a second greenhouse experiment, Zobiole et al. [95] raised GR soybeans of two cultivars (BRS242 RR1 and AG3539 RR2) on a Mexico silt loam soil. At the V2, V4 or V6 growth stages, soybeans were treated with Roundup WeatherMAX^®^ (Monsanto co.) at 800, 1200 or 2400 g a.e. ha^−1^. In herbicide-treated soybeans of both cultivars, leaf concentrations of several micronutrients (Zn, Mn, Fe, Cu, B) and macronutrients (N, P, Mg, K, Ca, Mg, S) were markedly reduced compared with controls at the R1 growth stage. In both experiments, and across all cultivars and soil types, reductions in leaf nutrient concentrations were accompanied by significant declines in the chlorophyll content of soybean leaves, as well as significant declines in the production of root and shoot biomass [94, 95]. Photosynthetic rate was also reduced in herbicide-treated plants of the medium and late maturity cultivar [97]. On the one hand, it is possible that accumulation of glyphosate (and/or its phytotoxic metabolite AMPA) in soybean sink tissues may be the direct cause of the observed chlorotic symptoms, since both glyphosate and AMPA have been shown to damage chlorophyll [89, 98–100]. For example, Gomes et al. [100] proposed that due to their chemical similarity, AMPA may compete with the essential amino acid glycine and thereby interfere with/inhibit its role in the biosynthesis of chlorophyll. Reduced photosynthetic efficiency may, in turn, reduce the energy allocated to root growth and restrict the capacity of the plant for nutrient uptake. Alternatively, it is possible that reduced leaf chlorophyll concentrations may have occurred as a secondary impact of the physiological inactivation of essential micronutrients (e.g. Mn, Mg) due to glyphosate–metal complexation.

Other authors have found GBH-induced reduction in nutrient uptake by GR crops to be strongly dependent on selected culture conditions [92, 96], as well as on differences between the herbicide formulations tested [20]. For example, when GR soybeans (cv. Valiosa RR) were supplied with sufficient Mn (0.5 µM) in hydroponic growth chambers, GBH treatments (Roundup^®^ UltraMax^®^) at the label-recommended dose reduced shoot Mn concentrations by ~ 50%, compared with controls. Root growth and elongation was also significantly reduced (~ 30%) in GBH-treated soybeans. No comparable responses occurred, however, in GBH-treated soybeans cultured with low Mn supply (0.1 µM). Likewise, leaf Zn concentrations were significantly reduced by treatments with Roundup^®^ Ultramax when soybeans (cv. Valiosa RR) were cultured in a sandy, acidic arenosol, but not on a calcareous loess sub-soil of luvisol [96]. Petter et al. [92] found that soil water conditions can strongly influence the degree to which nutrient accumulation is impaired by GBH exposure in GR soybeans. Under adequate moisture conditions, GBH applications (Roundup Ready; at doses of 1080 g and 1800 g a.e. ha^−1^) significantly reduced the accumulation of the macronutrients N, P, K, Ca and Mg and of micronutrients B, Mn, Zn and Fe in all three GR soybean cultivars tested (P98Y12RR, M9144RR and M9056RR) in a dose-dependent fashion. This was with the exception of one cultivar (M9144RR), in which N-accumulation was reduced, but not statistically significantly. In several, but not all cases, GBH-induced reduction in nutrient accumulation was significantly greater when plants were grown under conditions of soil water deficit [92].

Finally, a report by Zobiole et al. [101] found that GBH-induced inhibition of GR soybean metal uptake could be reversed by soil supplementation with a mixture of amino acids, including glycine. It is conventionally understood that GR crops should not be deficient in amino acids following treatment with a GBH, due to their resistance to glyphosate’s primary mode of action on plant EPSPS. However, the finding that addition of glycine reverses the effect of GBHs on GR crop mineral nutrition [101] suggests that GBHs may interfere with either the synthesis or function of glycine in GR soybeans. As suggested above with regard to chlorophyll synthesis [100], it seems possible that glyphosate and/or AMPA may be acting as a competitive analogue of glycine, potentially outcompeting the amino acid in biological sites and pathways. This could have knock-on effects for numerous plant physiological functions, including nutrient uptake.

Taken together, the above findings indicate that GBHs do have the potential to interfere with mineral nutrition in GR cultivars under some conditions, despite the engineered resistance of these cultivars to glyphosate’s intended mode of action on plant EPSPS. We repeat that herbicidal impacts appear to be strongly influenced by growth conditions such as soil properties. Interestingly, to our knowledge, the majority of papers which conclude that GBHs do interfere with GR crop nutrition used commercially formulated GBH products in their experimental trials (see above), whereas the majority of papers that do not identify any interference used pure glyphosate in their trials. The exceptions are two recent studies by Duke et al. [90, 91], both of which applied a commercial GBH, and both of which concluded that there was no significant effect of GBH treatment on the mineral content of GR soybeans. It is noteworthy that Duke et al. [6, 89, 90] pre-treated their experimental soils with other herbicides (*S*-metolachlor, pendimethalin and paraquat) which could have had unidentified influence on their findings, for instance by pre-empting further effects from GBHs. We propose that the contrasting conclusions put forward may be explained, at least in part, by the different herbicides used, since formulated products are likely to act differently in plants and soils to the active ingredient alone.

None of the mechanisms behind the observed interferences have been conclusively identified, although several authors cited glyphosate–metal chelation as a potential underlying factor [94–96]. Other possible factors include sub-lethal toxicity and impairment of soybean root physiology following accumulation of residual glyphosate and/or AMPA in root tissues [99, 100], and herbicide-induced disruption to rhizosphere-dwelling organisms associated with nutrient acquisition (see “Pathway I: disruptions to rhizosphere microbial ecology” section). Further investigation is clearly necessary to determine the relative contributions of the several, likely interrelated mechanisms which underlie the observed interference and their degree of expression in relevant agricultural contexts.

Nutrient restrictions of the kind observed in the above studies are likely to interfere with the physiological disease resistance of crops. If GBHs do indeed disrupt the nutrient status of crops significantly, then their disease-inducing impacts could be explained, at least in part, by Chaboussou’s [72] theory of *trophobiosis*. This, originally published in French in 1985, before the use of GBHs and GR crops, has been a widespread theory. Chaboussou showed, citing reviewed papers as well as his own experimental work, that disease-causing organisms are stimulated to proliferate when concentrations of free amino acids and reducing sugars are elevated in plant cells. These products are rich nutrient sources for bacterial and fungal phytopathogens, as well as insect pests. Any, even temporary, disruption of macromolecular synthesis results in elevated cellular concentrations of soluble amino acids and sugars, thus providing an environment where disease organisms are encouraged. Since various metal ions are involved in macromolecular (especially protein) synthesis, changes in their relative proportions are likely to be disruptive. This idea of *trophobiosis* would provide an overarching view of how nutrition affects disease, and hence account for one aspect of how GBHs may cause damage through their effects on the availability of nutrient metal ions.

## Conclusions

Cumulatively, the findings from the peer-reviewed literature presented in this review article counter the assumption that GBHs are innocuous when applied according to the manufacturers’ recommendations. The review sheds light on the extremely complex network of influences that glyphosate-based weed control techniques may have upon crops (of both GS and GR varieties) and upon their interactions with microbial phytopathogens.

It is important to recall, however, that considerable variability exists within these research findings, making it very difficult to reach generalised conclusions. We identified several factors that underlie this variability between different experimental studies including: (i) the properties of experimental soils and their mineral contents may differ greatly; (ii) the crop species and cultivars are often different; and (iii) commercial GBH formulations are formulated differently, frequently with unspecified ingredients (e.g. adjuvants) whose impacts are difficult to distinguish from those of the active ingredient. Numerous reports, including several of those cited above, show that formulated GBHs are orders of magnitude more active than glyphosate or its salts.

Nevertheless, one can draw a number of clear conclusions from the research collated here: (i) GBHs are shown to strongly undermine the innate physiological defences of GS crops by impairment of the shikimic acid pathway and by other biochemical effects; these render crops significantly weaker and more vulnerable to pathogenic attack; (ii) impairment of physiological disease defences has also been shown to occur in some GR cultivars, despite their engineered resistance to glyphosate’s primary mode of action; (iii) GBHs are shown to interfere with local microbial ecology upon their release to the rhizosphere. GBHs have the potential to promote phytopathogenic microbial species, through multiple inter-linked mechanisms (which include direct nutritional stimulation, as well as suppression of antagonists), enhancing their virulence and their damage to crops; and finally, (iv) the as yet incompletely understood influences of GBHs on the uptake and utilisation of nutrient metals by crops has the potential to further impair disease resistance. Importantly, the above deleterious impacts may occur in synergy with each other, and so compound and intensify each other’s damaging consequences.

The above conclusions are sufficient to conclude that glyphosate and its related products are not at all innocuous, in either GS or GR cropping systems. Rather, it is clear that the action of GBHs is not limited to the disruption of EPSPS in target plants, but can be far reaching. While we cannot conclude that the above impacts will occur in every case (we repeat that environmental conditions, crop cultivars and individual farming practices will strongly influence outcomes), and we can conclude that GBHs have the capacity to cause significant harm or impairment to the crops that they are employed to protect. Such a situation calls for urgent reconsideration of the wisdom of their widespread and intensive application in agriculture. These conclusions add to and complement the already substantial literature reporting the damaging effects of GBHs on animal life [4, 102].

Overall, we believe that the effects of GBHs are potentially wide ranging and are fundamentally linked to herbicide action. Our conclusions highlight the difficulty in developing a herbicide that is effective yet innocuous in relation to all non-target species and wider ecosystems. There exists a great need for collaboration between growers, regulators and industry to develop products, practices and policies that minimise the use of herbicides as far as possible and maximise their effectiveness when they are used, while facilitating optimised food production and security.

## Footnotes

1 That glyphosate can uncouple oxidative phosphorylation has been known for some time [62] and was quoted in the National Pesticides Information Centre Fact Sheet [7]. Recently, Swanson et al. [63] gave a detailed account of how glyphosate affects mammalian mitochondrial function, including by uncoupling.
